# Radiological Knosp, Revised-Knosp, and Hardy–Wilson Classifications for the Prediction of Surgical Outcomes in the Endoscopic Endonasal Surgery of Pituitary Adenomas: Study of 228 Cases

**DOI:** 10.3389/fonc.2021.807040

**Published:** 2022-01-20

**Authors:** Marta Araujo-Castro, Alberto Acitores Cancela, Carlos Vior, Eider Pascual-Corrales, Víctor Rodríguez Berrocal

**Affiliations:** ^1^ Neuroendocrinology Unit, Department of Endocrinology & Nutrition, Hospital Universitario Ramón y Cajal & Instituto de Investigación Biomédica Ramón y Cajal (IRYCIS), Madrid, Spain; ^2^ Department of Medicine, Universidad de Alcalá de Henares, Madrid, Spain; ^3^ Department of Neurosurgery, Hospital Universitario Ramón y Cajal, Madrid, Spain; ^4^ Department of Neurosurgery, Hospital HM Puerta del Sur, Madrid, Spain

**Keywords:** pituitary adenomas, invasive pituitary adenomas, Knosp classification, Hardy-Wilson classification, endoscopic endonasal transsphenoidal surgery

## Abstract

**Purpose:**

To evaluate which radiological classification, Knosp, revised-Knosp, or Hardy–Wilson classification, is better for the prediction of surgical outcomes in the endoscopic endonasal transsphenoidal (EET) surgery of pituitary adenomas (PAs).

**Methods:**

This is a retrospective study of patients with PAs who underwent EET PA resection for the first time between January 2009 and December 2020. Radiological cavernous sinus invasiveness was defined as a Knosp or revised-Knosp grade >2 or a grade E in the Hardy–Wilson classification.

**Results:**

A total of 228 patients with PAs were included. Cavernous sinus invasion was evident in 35.1% and suprasellar extension was evident in 74.6%. Overall, surgical cure was achieved in 64.3% of patients. Surgical cure was lower in invasive PAs than in non-invasive PAs (28.8% vs. 83.1%, *p* < 0.0001), and the risk of major complications was higher (13.8% vs. 3.4%, *p* = 0.003). The rate of surgical cure decreased as the grade of Knosp increased (*p* < 0.001), whereas the risk of complications increased (*p* < 0.001). Patients with Knosp 3B PAs tended to achieve surgical cure less commonly than Knosp 3A PAs (30.0% vs. 56.0%, *p* = 0.164). Similar results were observed based on the invasion and extension of Hardy–Wilson classification (stage A–C 83.1% vs. E 28.8% *p* < 0.0001, grade 0–II 81.1% vs. III–IV 59.7% *p* = 0.008). The Knosp classification offered the greatest diagnostic accuracy for the prediction of surgical cure (AUC 0.820), whereas the invasion Hardy–Wilson classification lacked utility for this purpose (AUC 0.654).

**Conclusion:**

The Knosp classifications offer a good orientation for the estimation of surgical cure and the risk of complications in patients with PAs submitted to EET surgery. However, the invasion Hardy–Wilson scale lacks utility for this purpose.

## Introduction

Pituitary surgery aims to eliminate excess hormone production in functioning pituitary adenomas (PAs), avoid or ameliorate tumor mass effects, preserve both pituitary function and adjacent nerve structures, and eliminate or reduce the risk of future recurrences (1). Nevertheless, the operative approach of PAs is guided by the size and location of the tumor and its relation to surrounding anatomical structures. This way, invasion of cavernous sinus is a known limiting factor in the achievement of complete surgical resection and could lead to a higher risk of postoperative surgical complications (2–4). Pre-surgical information about status of cavernous sinus invasion and the invasion of other parasellar structures is a key factor to planning surgery and for the estimation of the chances of surgical cure in PAs.

The earliest universally accepted, radiographic and operative classification of local invasion was proposed by Hardy et al. in 1976 (5) and later modified by Wilson in 1979 to distinguish between different grades of extrasellar extension (6). In 1993, Knosp et al. described the classical radiological classification of cavernous sinus invasion based on the relations of the PAs with the line between the supraclinoid internal carotid artery (ICA) and intra-cavernous ICA on coronal magnetic resonance imaging (MRI) (7). Several studies have found that the Knosp grade is a good predictor of surgical outcomes (4, 8, 9). Later studies have found that the revised-Knosp classification (3), which includes the differentiation between superior or inferior cavernous sinus compartment invasion in grades 3A and 3B, provides a better prediction of gross total resection and endocrine remission in functioning PAs (10). However, to the best of our knowledge, no previous studies have compared the Hardy–Wilson, Knosp, and revised-Knosp classifications for the prediction of surgical cure and complications in PA surgery and analyzed the correlation of these radiological scales with histological findings.

The aim of our study was to evaluate whether the radiological Knosp, revised-Knosp, and Hardy–Wilson classifications are good predictors of surgical outcomes in PAs, and which of these classifications have a greater predictive value for this purpose. Moreover, we have evaluated the correlation of these radiological classifications with histological findings. This information could be useful for surgical planning and for the estimation of the chances of surgical cure in PAs.

## Methods

### Patients

**Figure 1:**
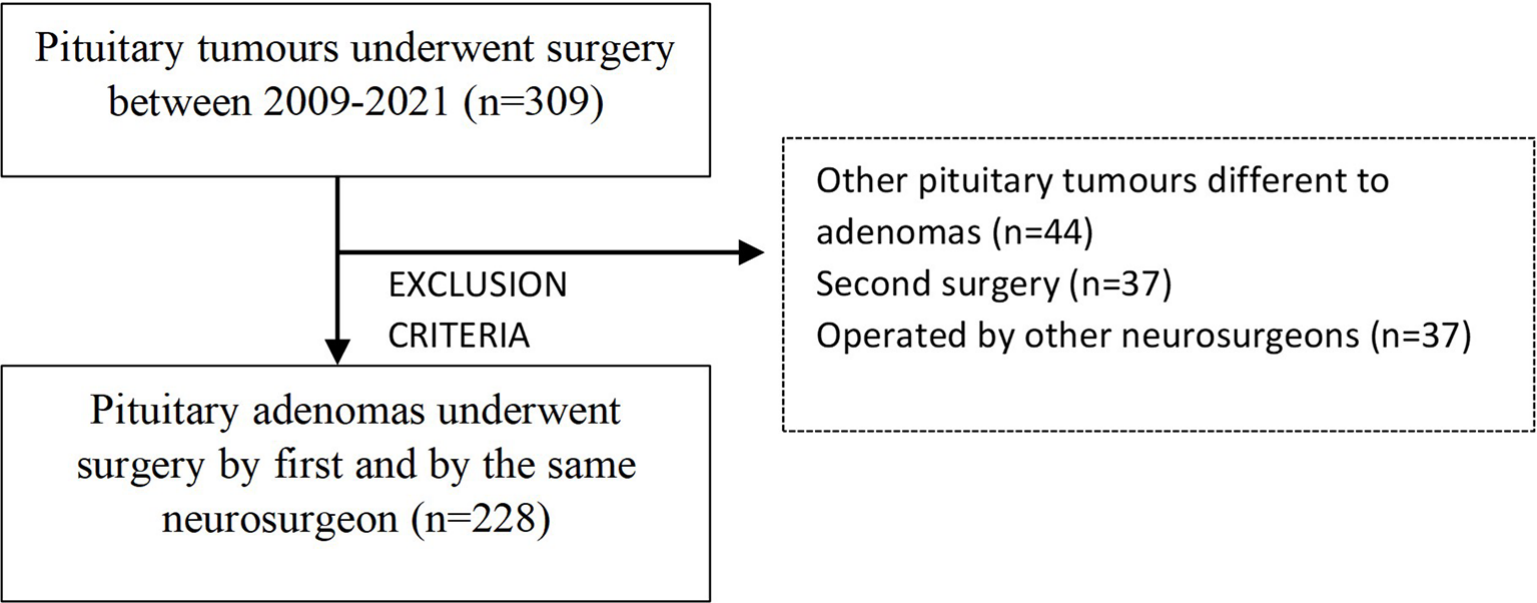
Study population.

A retrospective, two-center study was conducted. A total of 309 pituitary surgeries of patients with pituitary tumors operated between January 2009 and December 2020 at the Department of Neurosurgery of the Hospital Universitario Ramón y Cajal (HURC) and Hospital Universitario HM Puerta del Sur (HUPS) were identified. Clinical and radiological information was collected retrospectively between 2009 and 2012 (*n* = 45) and prospectively since January 2012 (*n* = 264). Inclusion criteria in the present study were as follows: (1) patients with available information about preoperative clinical, hormonal, and radiological data, and (2) pathology reports confirming PA diagnosis. Patients with Rathke’s cysts, craniopharyngioma, or pituicytoma diagnosis (*n* = 44), operated previously by the same or other neurosurgeons (*n* = 37), or operated by other neurosurgeons (*n* = 27) were excluded. A total of 228 patients met inclusion criteria and were enrolled (**Figure 1**) ***.*** The local Ethical Committee of the HURC and HUPS reviewed and approved this study (approval date: October 4, 2019, code: ACTA 372).

### Clinical and Hormonal Evaluation

The following demographic and clinical variables were included in our pituitary database: age, sex, diabetes mellitus, obesity, cardiovascular disease, hypertension, headache, visual impairment, and symptoms or signs of hypopituitarism, due to hormonal excess production. Visual involvement was defined as the presence of any degree of visual acuity compromise, from mild to severe visual acuity involvement and from partial to complete field conditions (9).

Hormonal assessment included preoperative measurement of 8am serum cortisol, ACTH, thyroid-stimulating hormone (TSH), free thyroxine (FT4), prolactin, follicle-stimulating hormone (FSH), luteinizing hormone (LH), insulin-like growth factor-1 (IGF-1), and total and free testosterone in males, as we have previously described (9). In those patients with 8am serum cortisol between 5 and 18 µg/dl, a 250-µg ACTH stimulating test was performed. The definitions of each pituitary hormone deficit were defined as we have previously described (11).

Biochemical cure was defined as normalization of urinary free cortisol in Cushing’s disease (12), as IGF-1 level in age- and sex-adjusted normal range and random GH value <2.5 ng/ml or GH value <1 ng/ml during an oral glucose tolerance test (OGTT) (13), as normalization of prolactin levels in prolactinoma, and as FT4 and FT3 normal levels in TSH-secreting PAs.

### Radiological Evaluation

Radiological evaluation was performed using an MRI of 1.5 T, GE 450w following our pituitary tumors protocol (1). The latero-lateral and craniocaudal diameters were assessed. PAs with maximum PA diameter <10, ≥10, ≥30 and <40, and ≥40 mm were defined as microadenomas, macroadenomas, very large, and giant PAs, respectively.

**Figure 2:**
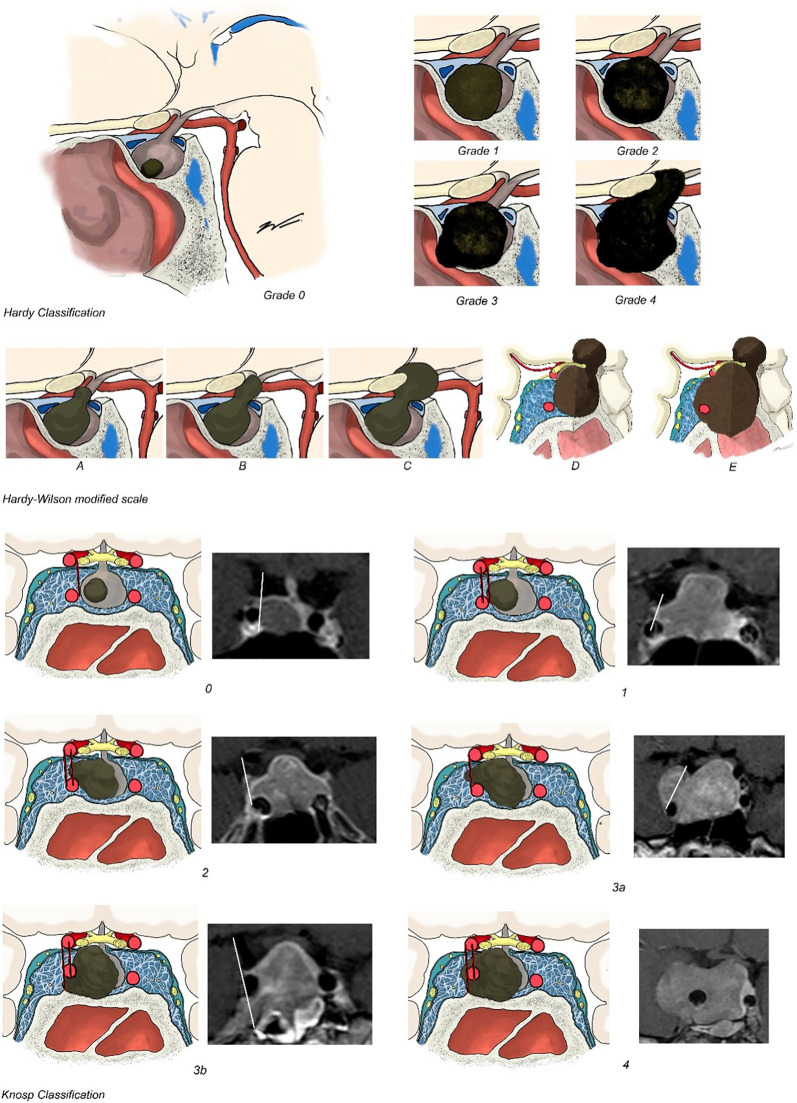
Hardy–Wilson and Knosp classifications. Hardy–Wilson classification considered the degree of sellar destruction: Grade 0 when the adenoma remains within the anatomical confines of the osteoaponeural sheath of the sella turcica; Grade I: the sella turcica is within normal limits in size or focally expanded and the tumor is <10 mm; Grade II: tumor ≥ 10 mm and the sella turcica is enlarged but the floor remains intact; Grade III: a local erosion or destruction of the floor; Grade IV when the entire floor of the sella is diffusely eroded or destroyed. Extrasellar extension according to Hardy–Wilson is divided in stage 0, with no suprasellar extension, A–C for progressive suprasellar extension. Knosp–Steiner classification considered: Knosp 0 when PA is medial to medial tangent; Knosp 1 if PA extends to the space between the medial tangent and the intercarotid line; Knosp 2 when PA extends to the space between the intercarotid line and the lateral tangent; Knosp 3 if PA extends lateral to the lateral tangent; and Knosp 4 with a complete encasement of intracavernous ICA. Knosp score 3–4 were considered as invasive PA. The revised-Knosp classification includes Knosp 3A when PA is above the intracavernous ICA into the superior cavernous sinus compartment and Knosp 3B when PA is below the intracavernous ICA into the inferior cavernous sinus compartment.

The Hardy–Wilson classification considered the degree of sellar destruction (grade) and extrasellar extension (stage) (14). Sellar destruction was divided into the following: Grade 0 when the enclosed adenoma is described as a tumor that remains within the anatomical confines of the osteoaponeural sheath of the sella turcica; Grade I: the sella turcica is within normal limits in size or focally expanded and the tumor is <10 mm; Grade II: tumor ≥ 10 mm and the sella turcica is enlarged but the floor remains intact; Grade III: a local erosion or destruction of the floor; Grade IV when the entire floor of the sella is diffusely eroded or destroyed, giving a characteristic “phantom sella” with all the boundaries barely visible. Extrasellar extension according to the Hardy–Wilson modified scale is divided into stage 0, with no suprasellar extension, A–C for progressive suprasellar extension (A: occupying cistern, B: recess of third ventricle obliterated, and C: third ventricle grossly displaced), and D–E grading parasellar extension (D: intracranial extension and E: cavernous sinus extension) (**Figure 2**).

**Figure 3:**
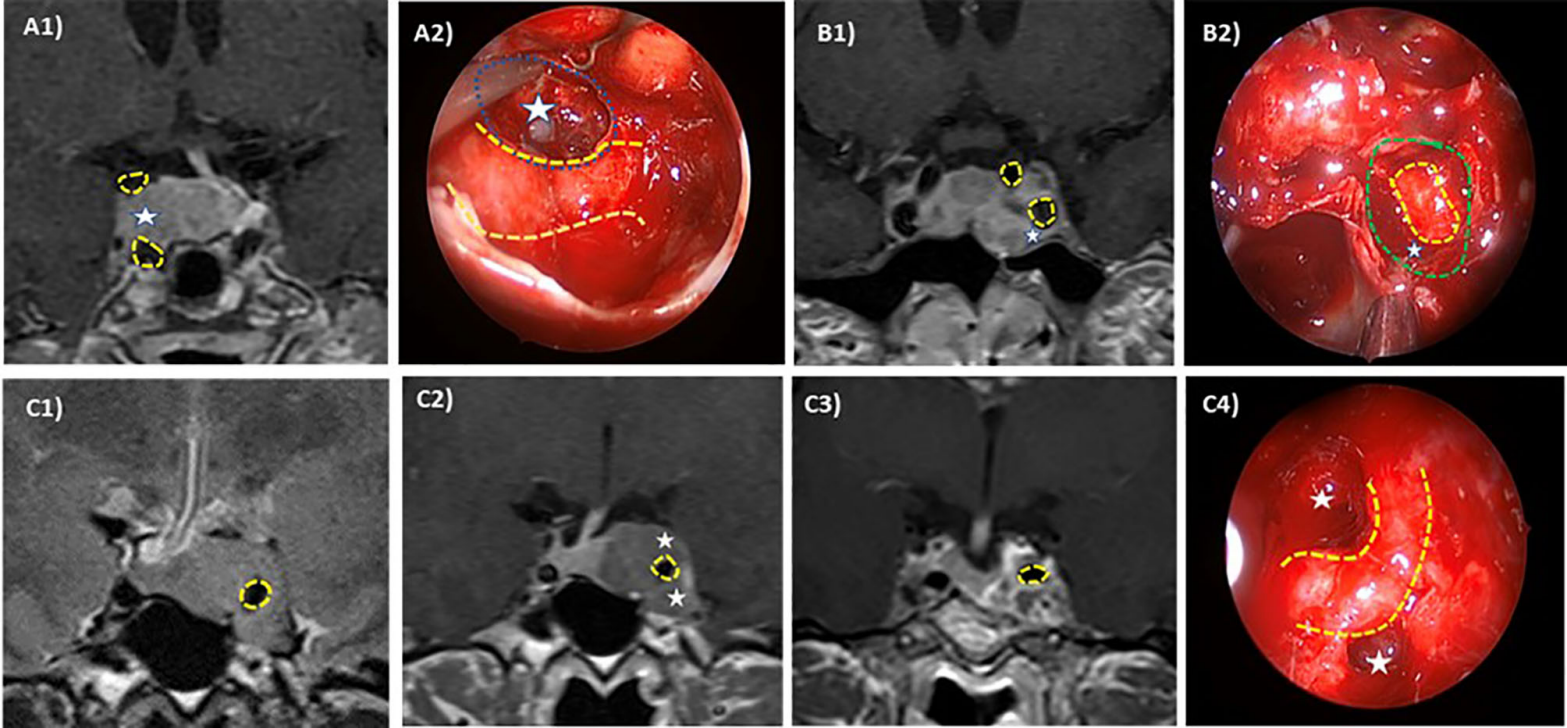
Intraoperative–radiologic correlation of cavernous sinus invasion in pituitary adenomas. ICA is highlighted in yellow dotted line and differently affected CS compartments are pointed out (white stars). Case 1: Right superior compartment invasion of the cavernous sinus (Knosp 3A) in an acromegalic patient. Preoperative MRI **(A1)** and intraoperative view through a 45° endoscope **(A2)** after tumor resection (left cavernous sinus medial wall resected in blue dotted line). The patient was cured after surgery. Case 2: Left Inferior compartment invasion of the CS in a resistant prolactin-secreting PA (Knosp 3B). Preoperative MRI **(B1)** and intraoperative view through a 0° endoscope **(B2)** after tumor resection showing anterior CS wall resection (green dotted line). Case 3: Complete cavernous sinus invasion (Knosp 4) in an acromegalic patient. Preoperative MRI in T2 sequences **(C1, C2)** shows a complete ICA encasement and the postoperative coronal MRI **(C3)** shows a near total resection. Intraoperative view through a 45° endoscope **(C4)** after tumor resection showing ICA and superior and inferior compartment. No surgical cure was achieved.

Cavernous sinus invasion was evaluated using the Knosp–Steiner classification based on coronal T1-weighted contrasted imaging (15): Knosp 0 when PA is medial to medial tangent; Knosp 1 if PA extends to the space between the medial tangent and the intercarotid line; Knosp 2 when PA extends to the space between the intercarotid line and the lateral tangent; Knosp 3 if PA extends lateral to the lateral tangent; and Knosp 4 with a complete encasement of intracavernous ICA. Knosp score 3–4 were considered as invasive PA. Moreover, radiological reports were reviewed to include the revised-Knosp classification (3, 5). The revised-Knosp classification includes 2 subtypes of grade 3: Knosp 3A when PA is above the intracavernous ICA into the superior cavernous sinus compartment and Knosp 3B when PA is below the intracavernous ICA into the inferior cavernous sinus compartment (**Figures 2** and **3**).

The extent of tumor resection (EOR) was classified into total (100%) or subtotal 70%–100% based on the 3–6 months postoperative MRI. Surgical cure is defined as total EOR in non-functioning PAs and by biochemical remission in functioning PAs.

### Surgical and Histological Evaluation

Pituitary surgery was performed by an experienced endoscopic pituitary surgeon (VB) with more than 300 endoscopic pituitary surgeries performed and an average of 35 pituitary surgeries/year in two high-volume centers (RyCUH and HMPSUH) during the last 10 years. The Endonasal Endoscopic Approach (EEA) was used in all surgeries included in this series. The approach included a binarial four-hand technique with wide anterior sphenoidotomy and partial posterior septectomy. In the cases with cavernous sinus invasion, an extended approach was performed. The macroscopic variables analyzed during pituitary surgery were tumor consistency (tumors difficult to remove with ring curettes and tumors that required sharp dissection, bipolar cautery, and/or surgical aspirator were termed hard tumors; the easily suckable were classified as soft tumors) and macroscopic information about dural, periosteal, or mucosal tissue invasion (defined as macroscopic invasiveness data). Complications have been divided into major [intrasellar bleeding (intra- or postoperative) requiring surgery, CSF fistula, meningitis, visual impairment with previously normal vision, new focal neurological deficit, carotid injury, stroke, or death] and minor (presence of diabetes insipidus, loss of the anterior pituitary hormonal axis, and medical complication).

The histological features evaluated were pituitary hormone immunostaining [ACTH, Prolactin and LH (polyclonal, Ventana), GH (clone A0570, Dako), TSH (clone 0042, Dako) and FSH (clone C10, Dako)] and the proliferation index based on Ki67 immunoexpression (clone MIB-1, Dako). Histological invasiveness was defined as invasion of sinus mucosa or adjacent bone in histopathologic sections.

### Statistical Analysis

The statistical analysis was performed using STATA.15. In the descriptive analysis, categorical variables were expressed as percentages and (absolute values of variable) quantitative variables were expressed as mean ± standard deviation. The normality assumption was studied with Shapiro–Wilk test. Student’s *t*-test was performed accordingly to compare differences in continuous parameters between two subgroups. The Chi-squared test was applied to compare categorical variables between independent samples. Kappa kohen index was used to evaluate the reliability between both classifications. ROC curves were performed to calculate the best predictive grade in the Knosp and Hardy classifications for surgical cure. The significance level was set at *p* < 0.05.

## Results

### Baseline Characteristics

In the last 11 years, 228 patients with PAs underwent transsphenoidal endoscopic endonasal PA resection for the first time by the senior author (VB). Non-functioning PAs represent 61.4% (*n* = 140) of the cohort, 22.4% (*n* = 51) had acromegaly, 11.0% (*n* = 25) Cushing’s disease, 4.8% (*n* = 11) prolactinoma, and one patient had a TSH-secreting PA. Forty patients were medically treated before surgery (8 patients with prolactinoma and 1 patient with acromegaly were treated with dopamine agonist, 24 patients with acromegaly were treated with somatostatin analogues, and 7 patients with Cushing’s disease were treated with adrenal steroidogenesis inhibitors (ketoconazole and/or metyrapone)], and no patients had a history of previous pituitary radiotherapy. Baseline patient’s characteristics are reported in **Table 1**.

**Table 1:** Patient and tumor characteristics of the study cohort at diagnosis.

Characteristic	Value
Age (years)	52.9 ± 15.4
Female sex	52.2% (*n* = 119)
Diabetes mellitus	13.2% (*n* = 30)
High blood pressure	30.7% (*n* = 70)
Obesity	10.5% (*n* = 24)
Heart disease	8.3% (*n* = 19)
Sleep apnea syndrome	7.9% (*n* = 18)
Headache	22.8% (*n* = 52)
Visual involvement	34.7% (*n* = 79)
Pituitary apoplexy	6.6% (*n* = 15)
Hypopituitarism	37.3% (*n* = 85)
Macroadenomas	86.3% (*n* = 196)
Knosp grade > 2/Hardy stage E	35.1% (*n* = 80)
Hardy grade > II (*n* = 173)	74.6% (*n* = 129)
Cranio-caudal diameter (mm)	20.7 ± 12.3
Latero-lateral diameter (mm)	19.0 ± 10.0
Total tumor resection in non-functioning PAs	69.3% (*n* = 88)
Biochemical cure in functioning PAs	65.9% (*n* = 58)
Postoperative permanent diabetes insipidus	4.8% (*n* = 11)
Cerebrospinal fluid leakage	5.3% (*n* = 12)
Postoperative new anterior pituitary deficits	13.3% (*n* = 19)
Hard consistency (*n* = 218)	28.9% (*n* = 63)

Invasion Hardy classification (sella destruction) was available only in 173 patients.

### Pre-Surgical Variables Associated With Radiological Invasiveness

Based on Knosp and extension Hardy classifications (stage), 35.1% (*n* = 80) presented cavernous sinus invasion. The Knosp grade distribution was as follows: grade 0 in 22.8% (*n* = 52), grade 1 in 15.4% (*n* = 35), grade 2 in 26.8% (*n* = 61), grade 3 in 16.2% (*n* = 37), and grade 4 in 18.9% (*n* = 43). Among the Knosp grade 3, 25 were grade 3A and 10 grade were 3B (in 2 patients, the images were not available for the classification in Knosp 3A and 3B) (**Figure 3**). Patients with invasive PAs presented larger tumors, which caused visual manifestations more commonly than non-invasive PAs (**Table 2**). Nevertheless, the higher prevalence of visual involvement in invasive PAs was related with their higher tumor size [adjusted OR: 1.0 (0.5–2.2)]. According to the Hardy invasion classification (*n* = 173), 5 were grade 0, 1 grade I, 38 grade II, 102 grade III, and 27 grade IV. Based on the Hardy extension classification, 148 were stage A–C (suprasellar extension) and 80 were stage E; 11 patients had intracranial extension of the PA although they were all considered grade E because of concomitant cavernous sinus invasion. As expected, when considered as dichotomic scales (Knosp 0–2 vs. 3–4 and Hardy stage A–C vs. E), there was an excellent accordance between the Knosp and extension Hardy classifications (kappa 1.00, global agreement 100%).

**Table 2:** Differences in clinical, hormonal, radiological, and histological features of invasive and non-invasive pituitary adenomas.

Variable	Knosp and extension Hardy classifications		
	Knosp ≤ 2/Hardy A–C (*n* = 148)	Knosp > 2/Hardy E (*n* = 80)	*p*-value
**Age (years)**	52.5 ± 15.3	53.5 ± 15.6	0.628
**Female sex**	54.7%	47.5%	0.297
**Headache**	19.6%	28.8%	0.116
**Visual defect**	24.3%	53.8%	<0.0001
**L-L diameter**	14.9 ± 7.4	26.5 ± 9.7	<0.0001
**C-C diameter**	16.2 ± 9.4	29.3 ± 12.7	<0.0001
**Hormonal deficit**	19.6%	21.3%	0.766
**Functioning PA**	41.9%	32.5%	0.164
**Histological invasion**	0.7% (*n* = 1)	7.5% (*n* = 6)	0.004
**Hard consistency**	23.6% (*n* = 33)	38.5% (*n* = 30)	0.020
**Ki67 > 4%**	7.4% (*n* = 11)	12.5% (*n* = 10)	0.207

L-L diameter, latero-lateral diameter; C-C diameter, cranio-caudal diameter; PA, pituitary adenoma.

### Knosp and Hardy Classifications: Correlation With Histological Invasiveness and Tumor Consistency

A clear association between the radiological Knosp and extension Hardy classifications and pathological invasiveness examination was found, as 7.6% of the Knosp 3–4 or stage E PAs had histological invasion compared to only one case of the non-invasive PAs (*p* = 0.004). Moreover, invasive PAs were more commonly of hard consistency than non-invasive PAs (**Table 2**).

### Knosp, Modified Knosp, and Hardy–Wilson Classifications: Impact on Surgical Outcomes

**Figure 4:**
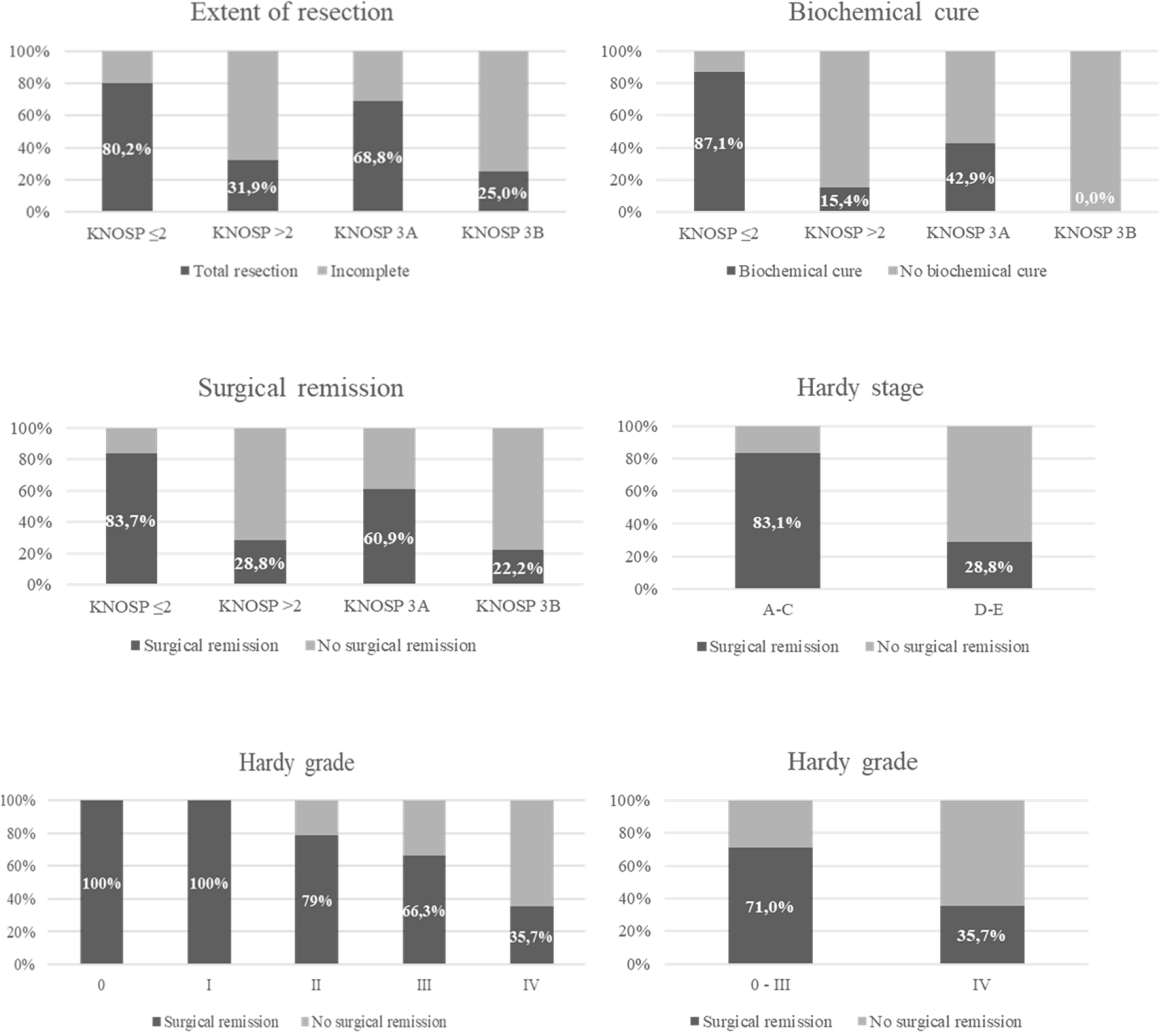
Surgical remission in functioning pituitary adenomas and total resection in non-functioning pituitary adenomas based on Knosp and Hardy classifications.

**Figure 5:**
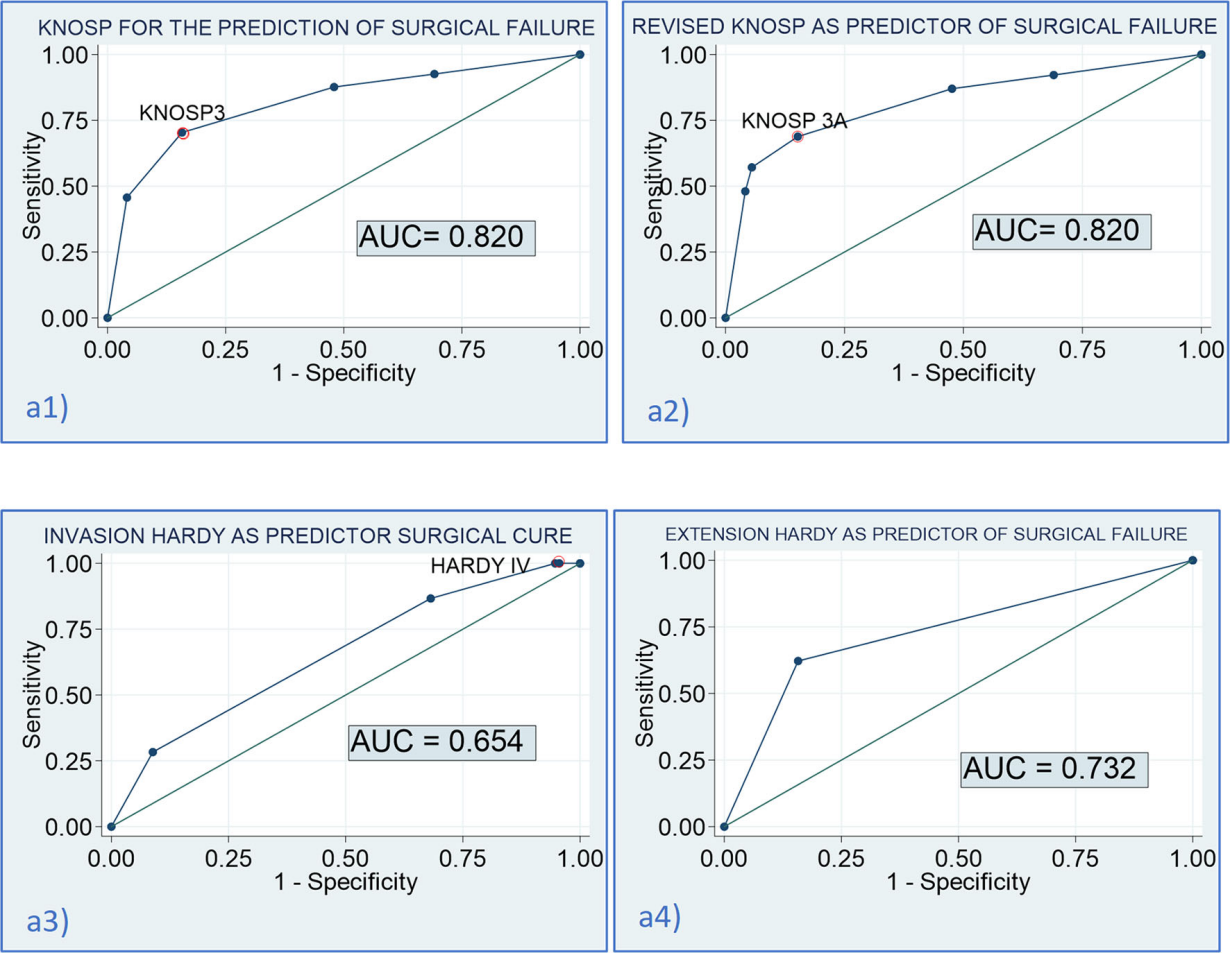
Diagnostic accuracy of the Knosp and Hardy scales for the prediction of surgical cure. (a1) AUC of the Knosp scale for the prediction of surgical failure: 0.820 [0.760–0.879]; optimal cutoff for the prediction of failure: Knosp 3 [sensitivity (Se) = 70.4% (59.7–79.2), specificity (Sp) = 84.2% (77.5–89.3); a2] AUC of the revised-Knosp scale for the prediction of surgical failure: 0.820 [0.760–0.882]; optimal cutoff for the prediction of failure: Knosp 3A [Se= 68.8% (57.8–78.1), Sp = 84.8% (78.1–89.8); a3] AUC of the invasion Hardy scale for the prediction of surgical failure: 0.654 [0.580–0.728]; optimal cutoff for the prediction of failure: Hardy IV [Se = 86.7% (75.8–93.1), Sp = 31.9% (24.0–40.9); a4] AUC of the extension Hardy scale (A–D vs. E) for the prediction of surgical failure: 0.732 [0.672–0.793]; optimal cutoff for the prediction of failure: Hardy stage E [Se = 62.2% (51.4–71.9), Sp = 84.2% (77.5–89.3)].

Overall, surgical cure was achieved in 64.3% of the patients: complete surgical resection in 69.3% (*n* = 88) of non-functioning PA and biochemical remission in 65.9% (*n* = 58) of functioning PAs (80% of Cushing’s disease, 66.7% of acromegaly, and 27.3% of prolactinomas and in the TSHoma). Complete surgical resection in non-functioning PAs and biochemical cure in functioning PAs were significantly higher in Knosp 0–2/Hardy stage A–C PAs than in Knosp 3–4/Hardy stage D–E PAs. Moreover, the risk of major complications and CSF leakage was lower in the group of non-invasive tumors (**Table 3** and **Figure 4**). No differences in the rate of surgical cure were observed between stage A and B (87.5% vs. 100%, *p* = 0.166), but patients of stage B achieved surgical cure more commonly than stage C patients (100% vs. 71.4%, *p* = 0.031). The rate of surgical complications was similar in stage A, B, and C (0%, 7.1% and 0%, *p* > 0.05). The higher risk chance of non-cure in radiological invasive PAs was independent of the tumor size [adjusted OR: 9.7 (4.7–20.2)], but the higher risk of major complications was related with the higher tumor size in these invasive PAs [adjusted OR: 2.0 (0.5–8.3)]. The overall rate of surgical cure decreased as the Knosp grade increased [MH Test for linear Trend: *χ* ^2^ = 66.8 (*p* < 0.001)], whereas the risk of complications increased [MH Test for linear Trend: *χ* ^2^(1) = 12.3 (*p* < 0.001)] (**Table 4** and **Figure 4**). A tendency to a lower rate of surgical remission was observed in Knosp 3B compared to 3A (30.0% vs. 56.0%, *p* = 0.164). Similar results were observed based on the invasion Hardy classification as 81.8% of Hardy 0–II vs. 59.7% in Hardy III–IV (*p* = 0.008) achieved surgical cure. Nevertheless, these differences disappeared after adjusting by tumor size [adjusted OR: 1.5 (0.6–3.8)] (**Table 5**). When comparing Hardy 0–III vs. IV, similar results were observed regarding surgical cure (70.6% vs. 37.0%, *p* = 0.001). However, in this case, the higher chance of surgical cure in grade 0–III persisted after adjusting by tumor size [adjusted OR: 2.8 (1.1–7.1)] (**Figure 4**). We found that the Knosp classifications considered as continuous scales (from grade 0 to 4) offered a greater diagnostic accuracy for the prediction of surgical cure [AUC 0.820 [0.760–0.879)], with the Knosp 3 and Knosp 3A being the ones that best predicted surgical failure (sensitivity 70.4%, specificity 84.2%). The AUC of the extension Hardy classification (grade A–C vs. grade D–E) was lower [AUC 0.769 [0.707–0.821)], especially the invasion Hardy classification (from I to IV) [AUC 0.654 [0.580–0.728)] (**Figure 5**).

**Table 3:** Surgical outcomes according to cavernous sinus invasion based on Knosp and revised-Knosp classifications.

Variable	Knosp classification			Revised-Knosp classification		
	Knosp ≤ 2 (*n* = 148)	Knosp > 2 (*n* = 80)	*p*-value	Knosp 3A (*n* = 25)	Knosp 3B (*n* = 10)	*p*-value
**Complete resection of NFPA**	80.2% (69/86)	31.9% (19/54)	<0.0001	68.8% (11/16)	33.3% (3/9)	0.087
**Biochemical cure of FPA**	87.1% (54/62)	15.4% (4/26)	<0.0001	33.3% (3/9)	0% (0/1)	0.490
**New hormonal deficits**	10.3% (10/97)	20.0% (9/46)	0.115	16.7% (2/12)	12.5% (1/8)	0.798
**Permanent DI**	4.7% (7/148)	5.0% (4/80)	0.911	0% (0/25)	10% (1/10)	0.109
**Any major complication**	3.4% (5/148)	13.8% (11/80)	0.003	8.0% (2/25)	10.0% (1/10)	0.849
**CSF leakage**	2.7% (4/148)	10.0% (8/80)	0.017	4.0% (1/25)	10.0% (1/10)	0.490
**Tumor recurrence**	6.1% (9/148)	5.0% (4/80)	0.913	8.0% (2/25)	0% (0/10)	0.357

**Table 4:** Surgical outcome based on the Knosp and revised-Knosp classifications.

Knosp	Surgical remission	Major complications	Risk of non-cure, 95% CI (considering Knosp 0 as reference)
0	86.5% (45/52)	0% (0/52)	1
1	88.6% (31/35)	2.9% (1/35)	0.97 [0.25–3.72]
2	77.1% (47/61)	6.6% (4/61)	2.23 [0.79–6.32]
3	46.0% (17/37)	8.1% (3/37)	4.59 [2.05–10.31]
3A	56.0% (14/25)	8.0% (2/25)	5.89 [1.84–12.82]
3B	30.0% (3/10)	10.0% (1/10)	17.5 [3.54–86.54]
4	14.0% (6/43)	18.6% (8/43)	46.25 [13.76–155.46]
Total	64.3% (146/228)	7.0% (16/228)	

The chances of non-cure increased as the Knosp grade increased [MH Test for linear Trend: χ^2^ = 73.1 (p = 0.0000)].

**Table 5:** Surgical outcome based on the invasion and extension Hardy classification.

	Surgical remission	Major complications
	**Hardy invasion**	
0	100% (5/5)	0% (0/5)
I	100% (1/1)	0% (0/1)
II	79.0% (30/38)	5.3% (2/38)
III	66.3% (67/101)	10.9% (11/101)
IV	35.7% (10/28)	7.1% (2/28)
	**Hardy invasion**	
A–D	83.1% (123/148)	3.4% (5/148)
E	28.8% (23/80)	13.8% (11/80)
**Total**	64.3% (146/228)	7.0% (16/228)

## Discussion

Since the standardization of the transsphenoidal approach and the use of a surgical microscope in this approach by Jules Hardy in 1968 (15–17), attempts were performed to predict the resectability of pituitary tumors through this approach, based on their preoperative radiological image. The first attempt with great international acceptance was performed by Hardy himself, considering the characteristics of bone remodeling produced by these lesions in the imaging tests available at the time (x-ray and CT). He described four types of local invasion around the sella (visible on x-ray and CT), depending on whether it was more or less remodeled (18). He also added a staging for the suprasellar extension (17), which Wilson later modified (6).

It would not be until the 1980s when MRI was progressively introduced as a neuroimaging technique (19). So, together with a greater diffusion of the transsphenoidal approach, E. Knosp and others, intuiting that the main prognostic factor was the invasion of the cavernous sinus, proposed a new classification based solely on its degree of invasion, according to the MRI image characteristics (7). The Knosp classification is the most widely accepted and used, although the Hardy–Wilson classification continues to be cited and described on numerous occasions. Thanks to the most recent development and improvements in endoscopic techniques, which allow for a better visualization of the parasellar structures, several studies have correlated the intraoperative involvement of the medial wall of the cavernous sinus with the Knosp radiological classification (20, 21). Despite significant discrepancies involving the Knosp classification system for PAs and its correlation with the invasion observed intraoperatively (3, 22), no other pre-surgical classification has been validated to date, nor has it been shown to correlate better with surgical results and clinical prognosis than Knosp classification.

Cavernous sinus invasion is one of the most unfavorable features of PAs. The most widely used classification was proposed by Knosp in 1993 and revised in 2015. In accordance with the reported data in several previous series, we found that patients with PAs with invasion of the cavernous sinus, both based on the Knosp and extension Hardy–Wilson classifications, had a lower chance of surgical cure and a higher risk of surgical complications. However, the Knosp and revised-Knosp classifications were the best for the prediction of surgical failure, with an area under de ROC curve of 0.820, with the Knosp grade 3 and Knosp 3A being the ones that best predict surgical failure (sensitivity 70.4%, specificity 84.2%). Nevertheless, despite these findings, we observed that all the radiological classifications were poor predictors of histological invasiveness, as only 7.5% of radiological invasive PAs had histological invasion. This finding highlights the fact that surgical inspection remains the gold standard to predict cavernous sinus invasion and that Knosp or modified Knosp classification presents a relevant number of false positives, as it has been previously reported (23).

We found that 28.8% of invasive PAs based on Knosp and extension Hardy classifications achieved surgical remission. This is in accordance with the data reported by other authors (10, 24, 25). Buchy et al. showed that gross total resection was negatively correlated with Knosp grade, while rates were 55.8% for grade 3A and 30.0% for grade 3B (10). Similarly, Micko et al. (24) showed that grade 3A PAs have a significantly lower rate of invasiveness of the medial cavernous sinus wall than grade 3B and 4 adenomas. Furthermore, infiltration of and fibrous tumor texture within the space of the cavernous sinus were found more frequently in grades 3B and 4. Consequently, grade 3A adenomas had a higher rate of endocrine remission/gross total resection (64%) than grade 3B (33%, *p* = 0.021) and grade 4 (0%, *p* < 0.001) PAs (24). Therefore, parasellar adenoma growth should be classified into grades 3A, 3B, and 4 for prediction of adenoma invasion and surgical considerations and outcomes. Moreover, the differentiation in Knosp low-grade (grades 1 and 2) and high-grade (grades 3 and 4) adenomas is important since the rates of achievement of complete resection for Knosp high-grade tumors are poor in comparison to those for low-grade adenomas. Thus, PA volume and cavernous sinus invasion, classified with the Knosp and revised-Knosp scales, are tumor features that can be used as resection predictor variables in PA surgery (25). It should be noted that in our series, we found that invasive PAs were 10 mm larger and caused clinical manifestation two times more than non-invasive PAs. Nevertheless, cavernous sinus invasion was a predictor of surgical failure independent of tumor size, with a probability of non-cure of almost ten times higher in invasive PAs than in non-invasive PA after adjusted by tumor size. Although the endoscopic transsphenoidal approach provides a panoramic vision inside the surgical area, a superior close-up of the anatomy, an improved working angle, and the lower probability of cure in invasive PAs could be related to the fact that gross total resection is usually more difficult in invasive PA than in non-invasive PA, especially if the tumor extends to the superior anterior clinoid process and posterior lateral ICA. In these cases, an expanded endonasal transcavernous approach should be considered (26). However, not only do anatomical characteristics play an important role in defining surgical planning and surgical goals, but also patient (age, comorbidities, symptoms) and tumor characteristics (acromegaly, Cushing disease or prolactin hypersecretion) are crucial, so the final decision must be individualized. Also, cavernous sinus invasion on preoperative imaging allows to predict higher surgical risk and lower cure rates, and this should be taken into account when giving informed consent to the patient. Different technical aspects have been described to improve the radiological accuracy of the diagnosis of cavernous sinus invasion, including 3-T MRI with the use of proton-density-weighted imaging or radiomics, among others (27).

Moreover, invasive PAs were more commonly of fibrous consistency than non-invasive PAs. Surgical outcomes seem to be associated with fibrous PA consistency. Up to 91% of adenomas are soft (meaning they are easily aspirated with conventional suction instruments), but approximately 10%–15% of patients will have tumors of fibrous consistency (requiring prior fragmentation with the use of a scalpel, forceps, or ultrasonic aspirators), associating a more significant number of incomplete resections and greater surgical risk (28–31). Fibrous PAs tend to be larger and invade neighboring structures, including the cavernous sinus. Some articles describe tumor remnants in cavernous sinus as fibrous, and this same consistency may be one of the causes of incomplete resection of the adenoma and ultimately implicating a higher number of recurrences (29, 24). It may be that the internal structure of the cavernous sinus itself, with trabeculae and ligaments, makes tumor removal difficult or a combination of this with the consistency of the adenoma (3, 32). A higher risk of hypopituitarism or hyponatremia, RR = 6.75 (95% CI 3.23, 14.07), has also been described in those patients with fibrous adenomas (33). Several authors have suggested that preoperative radiological features of the PA in the MRI could be useful to predict tumor consistency, especially when radiomic and machine learning on T2-weighted MRI (34), diffusion-weighted imaging (35), or MR elastography were employed (36).

In our study, Hardy extension classification (considered A–D vs. E stage) showed a good concordance with Knosp classification (grades 0–II vs. III–IV) as they are considered equivalent since they evaluate the same items. Moreover, although the rate of surgical remission decreased as the grade in the extension and invasion Hardy classification increased, we found that the diagnostic accuracy of the Hardy classification, especially the invasive (sella destruction) Hardy classification, was quite low for the prediction of surgical outcomes. It was a first approach with the diagnostic tools available at that time (x-rays and CT), where they use some radiological characteristics visible in those images (invasion and destruction of sellar floor bone). Nevertheless, some previous series found that suprasellar extension less than 10 mm was associated with favorable remission and resection rates (37). Similarly, Yang et al. (38) described expressly that PAs with intracranial extension had increased surgical complications and a lower rate of gross total removal, although not describing Knosp grades. On the other hand, other authors found that the remission rate was not associated with sellar floor erosion according to the Hardy–Wilson system of grading, neither with supra- and parasellar extension (39, 40). Thus, it seemed that Hardy–Wilson classification is not a reliable marker of surgical remission. Moreover, the increase of risk as invasion Hardy increased was related with a larger tumor size in these tumors. When we adjusted the risk by tumor size, invasion Hardy classification lost its ability to predict surgical failure. Considering these data, we can affirm that the only reliable characteristic that predicts resectability is the invasion of the cavernous sinus, which is better systematized and in a more detailed way in the Knosp classifications. In fact, the Hardy classification is currently rarely used as it is considered not very precise for the evaluation of PAs. However, despite Knosp classification being the most universally accepted classification for this purpose, other more sophisticated scores have been proposed recently, including the Zurich Pituitary Score, which is based on two quantitative measurements: the maximum horizontal tumor diameter and the minimum inter-carotid distance at the intracavernous horizontal C4 segment of the ICA, according to the Bouthillier classification. This classification is a simple and reproducible tool that reliably predicts surgical outcomes including the extent of resection, residual volume, and gross total resection of PA patients undergoing transsphenoidal pituitary surgery (41). Moreover, it has been demonstrated as an excellent inter-rater agreement in three different external cohorts (42).

The most outstanding data of our research were that Knosp and revised-Knosp classifications showed a high diagnostic accuracy to predict surgical outcomes. The AUC of these two classifications for the prediction of surgical cure was 0.82, and a positive tendency to higher rate of complications and a lower rate of surgical cure was observed as the grade of Knosp classification increased. We found that the prediction of surgical failure was independent of tumor size. Similar results regarding surgical cure were reported in acromegaly series (overall remission rate 84.7% vs. 69.1%, *p* < 0.001 in invasive PAs) (43), Cushing’s disease (77.1% in non-invasive vs. 53.0% in invasive PAs) (44), prolactinomas (95% in non-invasive vs. 20% in invasive PAs, *p* < 0.001) (45), and non-functioning PAs (92.5% non-invasive vs. 52.1% invasive PAs) (46). Although endoscopic surgery was considered to provide a better view for cavernous sinus invasion and superior structures and lesser nasal cavity injuries than microscopic surgery, the presence of invasion also seemed to clearly affect the surgical outcomes despite this supposed better visualization by the neurosurgeon. Moreover, the rate of surgical cure was as low as 28.8% in our cohort population. This figure is not lower than the one reported in previous endoscopic studies, with figures of even 5.9% having been described (47). Similar rates of approximately 30% have also been reported by other authors (48), and even a rate of 71% has been reported in a recent study evaluating the impact of an aggressive surgical approach that combined transsphenoidal transsellar and transmaxillary transpterygoidal approaches for the resection of grade 4 PAs (49). These differences in surgical remission rates probably depend on how conservative the surgical strategy regarding tumor resection was. Another important aspect to consider during pituitary surgery is complications. As previously reported (49, 50), we observed a higher proportion of surgical complications as higher Knosp grade of the PA. It is known that PAs invading the cavernous sinus are particularly surgically challenging due to their close proximity to critical neurovascular structures and their deep intracranial location.

In accordance with previous studies (8) and a recent meta-analysis (51), we found that patients with grade 3A showed a tendency to find a higher rate of surgical cure than those with grade 3B (56.0% vs. 30%). These results suggest that the revised-Knosp classification improves the accuracy of invasive PA diagnosis using surgical inspection. Nevertheless, the AUC of the ROC curve was equal to that of the classical Knosp classification. This could be related to the limited sample size of the Knosp 3 grade. In contrast, the Fang et al. meta-analysis (51) described that the modified Knosp had a remarkably higher AUC (0.91) than grades 3–4 (0.86) to predict cavernous sinus invasion, and thus probably for the prediction of surgical cure. Moreover, some studies have confirmed a high frequency of false positives in grade 3 in endoscopic series and recommended the addition of grades 3A and 3B into the existing parasellar classification (3, 52). We consider that although we did not find any differences in the diagnosis accuracy of the Knosp and revised-Knosp classification for the prediction of surgical cure, the differentiation between grades 3A and 3B is important as patients with grade A had a higher probability of surgical cure, which is more similar to grade 2 of Knosp, whereas the behavior of Knosp 3B is more similar to grade 4 of the classical Knosp. This information should be considered when surgery is being planned.

This study is not without limitations. One of them is related to its retrospective design, which must be considered when interpreting the results. Also, the operations performed in this series were also chiefly performed by a specialized skull base surgeon. Thus, the applicability of these results to centers with lower volume and experience performing endoscopic operations on invasive PAs may be limited.

## Conclusion

Cavernous sinus invasion remains a significant determinant limiting EOR in PA surgery. That is why Knosp and revised-Knosp classifications offer an excellent orientation for the estimation of surgical cure and the risk of complications in patients with PAs submitted to EET surgery. However, the invasion Hardy scale lacks utility for this purpose.

## Data Availability Statement

The raw data supporting the conclusions of this article will be made available by the authors, without undue reservation.

## Ethics Statement

The studies involving human participants were reviewed and approved by the Ethical Committee of the Ramón & Cajal Hospital. The patients/participants provided their written informed consent to participate in this study.

## Author Contributions

Conceptualization: MA-C. Methodology: MA-C. Formal analysis: MA-C. Data inclusion: VB and AC. Writing—original draft preparation: MA-C. Figure creation: CV. Writing—review and editing: MA-C, AC, CV, EP-C, and VB. All authors contributed to the article and approved the submitted version.

## Funding

This research and APC were funded by Fundación para la Investigación Biomédica del Hospital Universitario Ramón y Cajal (FIBioHRC). IRYCIS. Ctra. Colmenar Viejo km 9,100. 28034. Madrid, Spain. VAT ES-G-83726984.

## Conflict of Interest

The authors declare that the research was conducted in the absence of any commercial or financial relationships that could be construed as a potential conflict of interest.

## Publisher’s Note

All claims expressed in this article are solely those of the authors and do not necessarily represent those of their affiliated organizations, or those of the publisher, the editors and the reviewers. Any product that may be evaluated in this article, or claim that may be made by its manufacturer, is not guaranteed or endorsed by the publisher.
